# Extracorporeal Membrane Oxygenation Cannulation Site Affects Coronary and Cerebral Perfusion When Combined With Intra-Aortic Balloon Pump

**DOI:** 10.1097/MAT.0000000000002454

**Published:** 2025-05-16

**Authors:** Vincent Sibut-Pinote, Philippe Reymond, Mustafa Cikirikcioglu, Karim Bendjelid, Christoph Huber

**Affiliations:** From the * Charles Hahn Hemodynamic Propulsion Laboratory, Medical Faculty, University of Geneva, Geneva, Switzerland; † Division of Cardiovascular Surgery, Department of Surgery, University Hospitals and Medical Faculty of Geneva, Geneva, Switzerland; ‡ Department of Anesthesiology, Pharmacology and Intensive Care, Geneva Hemodynamic Research Group, University Hospitals and Medical Faculty of Geneva, Geneva, Switzerland.

**Keywords:** ECMO, IABP, coronary blood flow, cerebral blood flow, renal blood flow, *in vitro* mockup circuit, low cardiac output syndrome

## Abstract

The use of intra-aortic balloon pump (IABP) alongside venoarterial extracorporeal membrane oxygenation (VA-ECMO) in critically ill patients presenting refractory cardiogenic shock raises questions regarding its impact on organs perfusion. This *in vitro* study aimed to examine the combined effects of IABP and VA-ECMO on coronary, cerebral, and renal perfusion, particularly considering the choice of arterial cannulation site. A mock circuit with a pulsatile pump was used to simulate different scenarios with increasing severities of low cardiac output syndromes treated by concomitant IABP and VA-ECMO support. Flow rates were measured using ultrasonic flowmeters. Each scenario was tested with two different VA-ECMO outflow access sites: femoral and axillary arteries, at heart rates of 60 and 100 bpm. Results showed that concomitant use of IABP with VA-ECMO in the axillary artery increases more significantly cerebral and coronary flow rates compared to femoral access in intermediate and severe shock. Nevertheless, renal perfusion appeared to be more negatively affected in this configuration. In summary, employing IABP alongside axillary VA-ECMO enhances cerebral and coronary flow but may compromise renal perfusion. Shock severity, heart rate, and cannulation site should be considered for a tailored approach. Future investigations using sophisticated autoregulated systems are needed to confirm these observations.

Patients with low cardiac output syndrome (LCOS) may require circulatory support to ensure proper organ perfusion and oxygenation. The intra-aortic balloon pump (IABP) and the venoarterial extracorporeal oxygenation membrane (VA-ECMO) are two devices commonly used in the management of these patients. Although these two devices have different mechanisms of action, their concomitant use has been reported in numerous clinical studies, with conflicting results. Several meta-analyses have demonstrated the benefits of the VA-ECMO/IABP combination for patients with cardiogenic shock,^1–3^ but another meta-analysis by Cheng *et al.*^4^ showed no advantage over VA-ECMO alone. In addition, several arterial cannulation sites for the VA-ECMO have been reported in the literature: femoral and subclavian or axillary cannulation.^5–7^ These two cannulation sites offer retrograde and anterograde flow respectively to the thoracic and abdominal aorta, which may influence the effects of a VA-ECMO/IABP combination. Although the anterograde flow of axillary arterial cannulation could have benefits for the patient, for example, by facilitating ambulation^8^ or reducing the risk of embolization,^5^ a recent study showed that this site should be reserved for patients with compromised femoral vascular access.^7^

The present new *in vitro* study follows on our previous publication, in which we showed that the concomitant use of IABP and VA-ECMO *via* the femoral arterial cannulation increased mean coronary flow. The aim of this new study is to analyze the hemodynamic effects of this combination, notably on cerebral and renal circulation, as well as the influence of the arterial cannulation site on the combination with IABP.

## Materials and Methods

The mock circuit is based on the one used during the previous study,^9^ to which we have made several modifications, detailed in this chapter, to meet the previous limitations.

### Low Cardiac Output Syndrome Scenarios

We simulated several LCOS scenarios of varying degrees of severity. For each VA-ECMO configuration (femoral *versus* axillary cannulation), we have taken measurements under “baseline” conditions: pump output of 5 L/min, without compensation by VA-ECMO. An “intermediate” scenario with a pump flow of 3 L/min and an ECMO outflow of 2 L/min. A “severe” scenario with a pump flow of 2 L/min and an ECMO outflow of 3 L/min. In each of these simulations, we took measurements at heart rates of 60 and 100 beats per minute (bpm), without IABP and with IABP in 1:1 mode (balloon inflating with each diastole) at 100% inflation amplitude.

### Systemic Circulation Modeling

**Figure 1.**
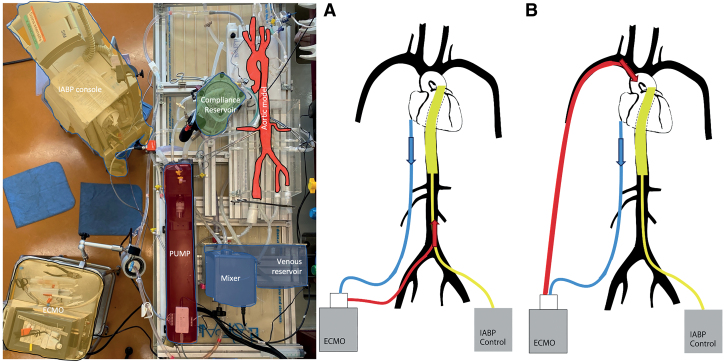
Mock circuit set-up with the different components. On the right, the two arterial cannulation sites, femoral (**A**) and subclavian (**B**), offering flows represented by the red arrows (retrograde and anterograde, respectively). ECMO, extracorporeal membrane oxygenation; IABP, intra-aortic balloon pump.

We used the same silicone circuit as in our previous publication (model ref T-S-N-009+; Elastrat, Geneva, Switzerland). To prevent the formation of air bubbles when testing at a heart rate of 100 bpm, and to improve the quality of aortic flow measurements, a rigid plastic grid was placed in the compliance reservoir tank. We used a pulsatile pump (Superpump; ViVitro Labs, Inc., Victoria, BC, Canada) to simulate cardiac function at the circuit inlet. Positioning the pump in line with the aortic root was the configuration with the fewest disturbances flow and pressure measurements (Figure 1).

### Coronary Circulation Modeling

**Figure 2.**
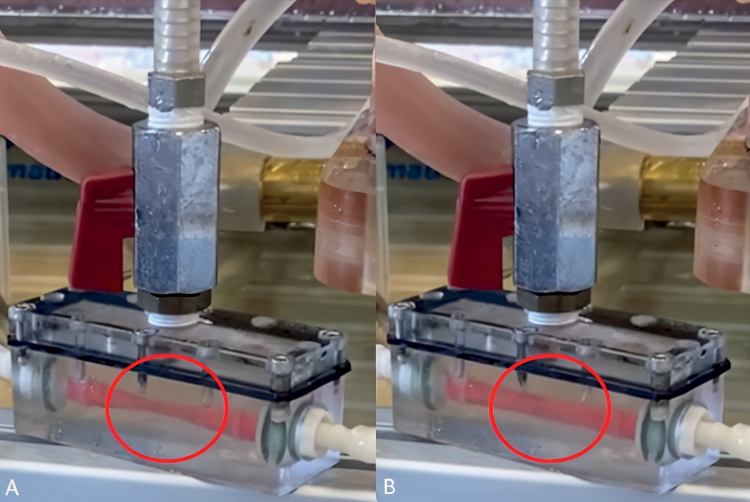
Coronary model in a sealed chamber connected in parallel to the pump, allowing wall compression during systole (**A**) and relaxation during diastole (**B**). Complete cycles are showed in Supplementary Video 1.

**Figure 3.**
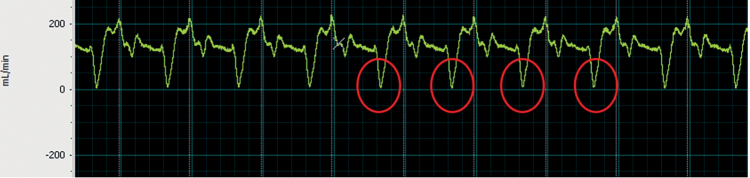
Coronary flow in ml/min curves obtained with the circuit (LabChart 8 pro screenshot). Circled in red, the drop in flow during systole, obtained by extrinsic compression of the coronary model in synchrony with the pump.

We have modified the coronary artery model to approximate as closely as possible the physiologic coronary flow waveforms. We based our model on Geven *et al.*^10^ We used a silicone tube with low radial resistance placed in a sealed chamber, connected to the pump compartment representing the left ventricle (LV) with a rigid tube filled with water (Figures 2 and 3). This system allowed us to simulate radial compression of the coronary arteries by the myocardium during systole.^11^

### Cerebral Circulation Modeling

To model cerebral circulation, a tube was placed in series with the systemic circulation circuit. Using a Hoffmann clamp and an ultrasonographic flowmeter, we were able to regulate cerebral flow to a constant value of approximately 12.5 ml/s, which represents between 700 and 800 ml/min under baseline conditions.^12^

### Renal Flow Modeling

Our silicone aortic model features the main arteries originating from the aortic arch and the abdominal aorta (Figure 1). We were thus able to measure flow in one of the renal arteries. This flow was regulated and measured in the same way as cerebral flow at around 300 ml/min.

### Blood Modeling

The rheological properties of blood were approximated with a mixture of water and glycerin, in proportions of 60% and 40%, respectively.^9^ A mixer was placed in the reservoir to achieve optimum fluid homogeneity. Fluid samples were taken between the different measurement periods to check the density of the mixture and ensure reproducibility.

### Electrocardiogram Modeling

In our previous study, the timing of inflation and deflation of the IABP was calibrated on pressure curves obtained from the pressure catheter at the tip of the intra-aortic balloon. Due to the large change in pressure curves when using the balloon, we observed a loss of inflation triggering after only a few cardiac cycles, making our measurements difficult to interpret and, above all, unreproducible. To improve this aspect, we synchronized the IABP with an electrocardiogram (ECG). In synchronization with the pump displacement curve, a signal (1V voltage) mimicked the QRS complex on the IABP console. As reported in the literature, we set an offset of 50 ms between the ECG signal representing the QRS and the start of systole.^13^

### Extracorporeal Membrane Oxygenation

We used a VA-ECMO (Maquet GetingeGroup, Wayne, NJ), without the oxygenation membrane. The arterial cannula was placed in the femoral or axillary artery. We performed the same data series for both cannulation sites.

### Intra-Aortic Balloon Pump

The catheter for the intra-aortic counterpulsation balloon (MEGA 8 Fr-50cc, Datascope Corp, Fairfield, NJ, Maquet GetingeGroup) was placed in the thoracic aorta *via* an introducer in the left iliac artery. The balloon was connected to a Maquet CS100 console. We used the balloon in manual mode, triggered by the ECG source and inflated at 100% amplitude. For each scenario, an initial series of data was collected without balloon, then with IABP in 1:1 mode. Balloon timing was set manually before each measurement, in 1:2 mode over several operating cycles.

### Pressure and Flow Measurements

The same pressure and flow measurement equipment as in the previous study was used: an arterial pressure transducer (Transpac IT monitoring kit; ICU Medical, San Clemente, CA) at the level of the aortic arch, Transonic ME19PXN and ME4PXN (Transonic System Inc., Ithaca, NY) in the aortic root and coronary artery respectively, connected to the Transonic T402-TB meter. A flow sensor (SONOFLOW CO.55/120 V2.0 ultrasonic flow sensor; Sonotec, Halle, Germany) was used to measure renal and cerebral flow rates.

### Data Collection and Analysis

Data were collected using the PowerLab 8/35 acquisition card (ADInstruments, Dunedin, New Zealand) with a sampling rate of 1 kHz. For each measurement, we averaged 10 consecutive pump cycles at each change of scenario. All calculations are based on these averages.

## Results

**Figure 4.**
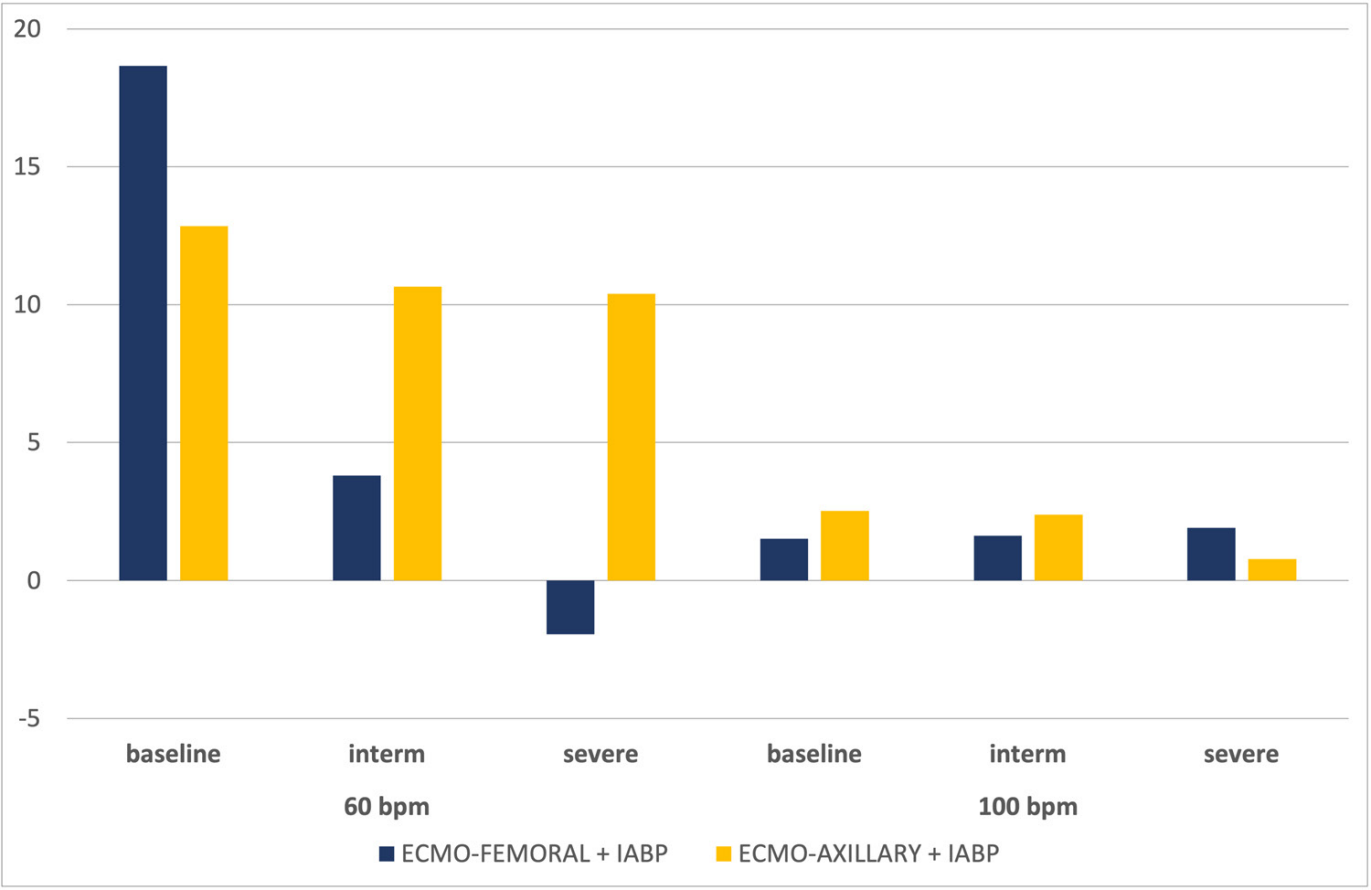
Variation in % of coronary flow for each scenario with the addition of IABP in 1:1 mode compared with ECMO alone, as a function of ECMO arterial cannulation site (blue: femoral, yellow: axillary), at 60 and 100 bpm. ECMO, extracorporeal membrane oxygenation; IABP, intra-aortic balloon pump.

**Figure 5.**
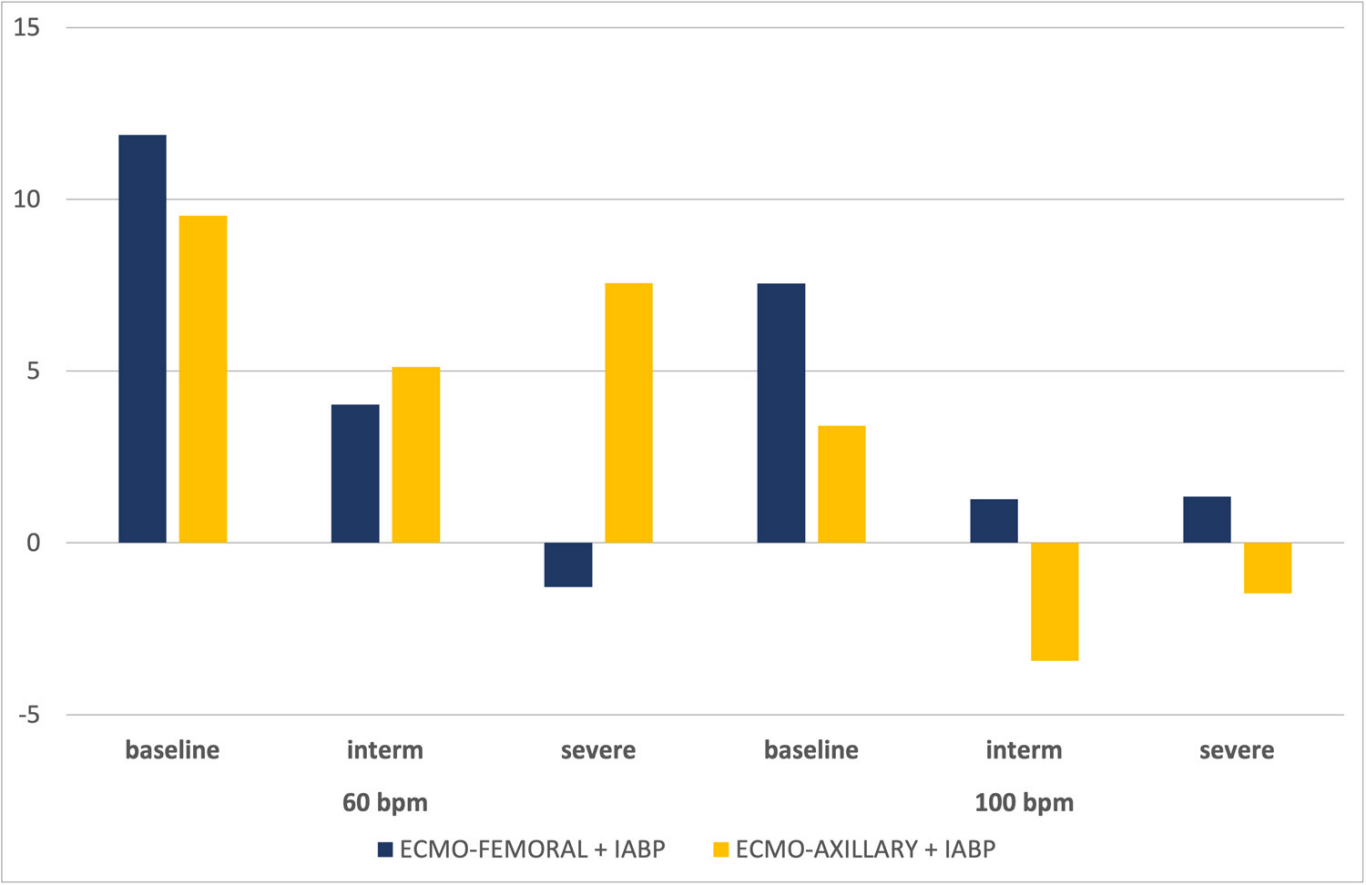
Variation in % of cerebral flow for each scenario with the addition of IABP in 1:1 mode compared with ECMO alone, as a function of ECMO arterial cannulation site (blue: femoral, yellow: axillary), at 60 and 100 bpm. ECMO, extracorporeal membrane oxygenation; IABP, intra-aortic balloon pump.

**Figure 6.**
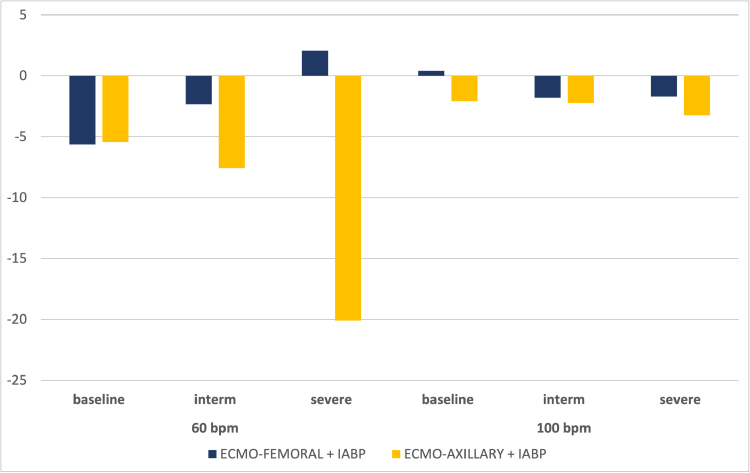
Variation in % of renal flow for each scenario with the addition of IABP in 1:1 mode compared with ECMO alone, as a function of ECMO arterial cannulation site (blue: femoral, yellow: axillary), at 60 and 100 bpm. ECMO, extracorporeal membrane oxygenation; IABP, intra-aortic balloon pump.

In this study, we examined the hemodynamic effects of the concomitant use of IABP and VA-ECMO, comparing two sites of arterial cannulation of VA-ECMO: femoral (VA-ECMO FF) and axillary (VA-ECMO FA). The measurements were carried out under baseline conditions, then in different scenarios of severity of the VA-ECMO-compensated LCOS, and at two different heart rates, for each organ of interest. The results are presented in Tables 1 and 2 and Figures 4–6.

**Table 1.** Data Obtained With Venoarterial ECMO in Femoro-Femoral Canulation, for Each Low Cardiac Output Syndrome Scenario at 60 and 100 bpm

Scenario	IABP	Q ECMO (L/min)	Q Coronary (ml/min)	Q Cerebral (ml/min)	Q Renal (ml/min)
Baseline 60 bpm	Off	0	126	715	235
	1:1		(+19%)	(+12%)	(−6%)
Intermediate 60 bpm	Off	2	108	671	228
	1:1		(+4%)	(+4%)	(−2%)
Severe 60 bpm	Off	3	98	662	218
	1:1		(−2%)	(−1%)	(+2%)
Baseline 100 bpm	Off	0	147	661	271
	1:1		(+2%)	(+8%)	(0%)
Intermediate 100 bpm	Off	2	142	663	264
	1:1		(+2%)	(+1%)	(−2%)
Severe 100 bpm	Off	3	137	634	253
	1:1		(+2%)	(+1%)	(−2%)

Mean coronary, cerebral, and renal flow rates are expressed in ml/min. Variation with intra-aortic balloon pump is expressed in %.

bpm, beats per minute; ECMO, extracorporeal membrane oxygenation; IABP, intra-aortic balloon pump; Q, flow.

**Table 2.** Data Obtained With Venoarterial Extracorporeal Membrane Oxygenation in Femoro-Axillary Cannulation, for Each Low Cardiac Output Syndrome Scenario at 60 and 100 bpm

Scenario	IABP	Q ECMO (L/min)	Q Coronary (ml/min)	Q Cerebral (ml/min)	Q Renal (ml/min)
Baseline 60 bpm	Off	0	92	724	254
	1:1		(+13%)	(+10%)	(−5%)
Intermediate 60 bpm	Off	2	86	672	211
	1:1		(+11%)	(+5%)	(−8%)
Severe 60 bpm	Off	3	83	637	240
	1:1		(+10%)	(+8%)	(−20%)
Baseline 100 bpm	Off	0	153	715	316
	1:1		(+3%)	(+3%)	(−2%)
Intermediate 100 bpm	Off	2	145	668	257
	1:1		(+2%)	(−3%)	(−2%)
Severe 100 bpm	Off	3	146	635	250
	1:1		(+1%)	(−1%)	(−3%)

Mean coronary, cerebral, and renal flow rates are expressed in ml/min. Variation with intra-aortic balloon pump is expressed in %.

bpm, beats per minute; ECMO, extracorporeal membrane oxygenation; IABP, intra-aortic balloon pump; Q, flow.

### Coronary Circulation

The set-up we used enabled us to obtain coronary flow curves like those obtained by Geven *et al.*,^10^ with a drop in coronary flow during systole, induced *in vivo* by the tension exerted by the myocardium on the coronary arteries.^11^ The curves obtained in our study are shown in Figure 3.

#### Influence of heart rate

Our data show that the addition of IABP to VA-ECMO increases mean coronary flow, more significantly at a heart rate of 60 than 100 bpm. At 60 bpm, an increase of +19% under baseline conditions was observed, compared with +2% at 100 bpm. In the intermediate scenario, we observed an increase of +4% and +2% at 60 and 100 bpm, respectively. In the case of severe LCOS, a variation of −2% and +2% was observed at 60 and 100 bpm, respectively.

#### Influence of cannulation site

At 60 bpm, the cannulation site influenced the variation in mean coronary flow. In the intermediate scenario, the addition of IABP to VA-ECMO FF increased mean flow by +4%, compared with +11% with VA-ECMO FA. In the severe scenario, mean coronary flow decreased by −2% in the ECMO-FF configuration *versus* a +10% increase with ECMO-FA. At 100 bpm, the differences between the two cannulation sites were less pronounced with the addition of IABP (results are presented in Tables 1 and 2).

### Cerebral Circulation

#### Influence of heart rate

As with coronary circulation, cerebral circulation is positively affected by the addition of IABP to VA-ECMO. At 60 bpm in baseline conditions, IABP in 1:1 mode increased cerebral output by +12%, compared with +8% at 100 bpm. In the intermediate scenario, the increase in flow with IABP was +4% at 60 bpm *versus* +1% at 100 bpm. In the severe scenario, we observed a decrease of −1% at 60 bpm *versus* an increase of +1% at 100 bpm.

#### Influence of cannulation site

At 60 bpm, in the intermediate scenario, the flow variation was similar, at +4% and +5%, respectively, in FF and FA. In the severe scenario, an increase of 8% was observed with IABP in FA, compared with a decrease of −1% in FF. At 100 bpm, the FF configuration increased mean flow by +1% in both scenarios and decreased when VA-ECMO was in FA (−3% in intermediate *versus* −1% in severe).

### Renal Circulation

#### Influence of heart rate

Under baseline conditions, renal output is negatively impacted by the action of IABP in 1:1 mode. We observed a −6% drop in blood flow at 60 bpm, while no change was observed at 100 bpm. In an intermediate LCOS, we observed the same −2% decrease in mean flow at both 60 and 100 bpm. In the severe case, a +2% increase in flow was observed in FF at 60 bpm, compared with a −2% decrease at 100 bpm.

#### Influence of cannulation site

Cannulation site showed significant variation differences in mean renal flow, especially at 60 bpm. Indeed, a −8% decrease was observed in the intermediate scenario with FA cannulation, compared with a −2% decrease with FF cannulation. In the severe case, at 60 bpm, the decrease in mean renal flow was −20% in FA *versus* a slight increase of +2% in FF. At 100 bpm, the change in mean renal flow was between −2% and −3% in FF or FA, whether in intermediate or severe LCOS.

## Discussion

The ECMO is a hemodynamic support device used in refractory shock. Using a venous cannula, the patient’s blood is drawn close to the right atrium into the inferior vena cava (Figure 1). It is then fed to a membrane for oxygenation. The oxygenated blood is then reintroduced into the patient’s circulation *via* an arterial cannula. This cannula can be positioned centrally (*e.g.*, during heart surgery) or peripherally (axillary, subclavian, or femoral). The intra-aortic counter pulsation balloon pump is an invasive hemodynamic support device. Using a balloon positioned in the thoracic aorta and synchronized with the patient’s ECG, the balloon can inflate during diastole and deflate during systole. During diastole, balloon inflation increases coronary perfusion. During systole, deflation produces a suction effect, reducing LV afterload and thus increasing ejection volume.

In intensive care practice, VA-ECMO is often cannulated into the femoral artery. This produces retrograde flow for the heart, increasing LV afterload and understandably LV end-diastolic pressure (LVEDP). In this regard, during cardiogenic shock VA-ECMO is beneficial to all organs perfusion but could be harmful to a fragile heart with LV dysfunction and low ejection fraction. Indeed, in this setting, under ECMO-VA the native cardiac function is impeded, cardiac oxygen consumption increased, and the accompanying pulmonary congestion may complicate the whole.

The concomitant use of the unloading effect of IABP theoretically makes it possible to reduce afterload on the LV, thereby improving ejection volume. It also increases coronary perfusion.^14^ Farag *et al*.^15^ have shown that the effectiveness of IABP in reducing LVEDP and left ventricular end-diastolic volume (LVEDV) depends on LV contractility. The lower its function, the greater the efficacy of IABP. The concomitant use of these two hemodynamic support devices is frequently reported in the literature,^14^ and clinical data are encouraging. Two meta-analyses from 2019 to 2022 on 4,576 and 2,573 patients, respectively, show that a combination of IABP/VA-ECMO improves patient survival compared with VA-ECMO alone. In the first study,^2^ neurologic, gastrointestinal, or lower limb complications were not more frequent in the IABP group. In the second study,^3^ there was no increase in bleeding, infection, or length of stay.

### Effects of Cannulation Site

Our results show that the site of arterial cannulation of the VA-ECMO is an important parameter influencing flow rates to the various organs. Femoral arterial cannulation provides retrograde flow, which is re-injected caudally to the IABP, whereas subclavian/axillary cannulation provides anterograde flow, which arrives cranially to the IABP. Our hypothesis is that arteries arising from the aortic arch (coronary, brachiocephalic trunk, left common carotid, and left subclavian) benefit from uninterrupted flow through the balloon in diastole when arterial cannulation is axillary. This hypothesis is consistent with the results we observe, mainly when LCOS is severe: cerebral and coronary flows are increased more significantly by IABP with anterograde flow. The renal arteries, distal to the balloon in this FA configuration, see their flow more strongly reduced with IABP than when the cannulation is FF. Several studies have investigated the different sites of arterial cannulation for VA-ECMO. Regarding axillary cannulation for VA-ECMO, data are not always concordant. A study by Moussa *et al*.^7^ involving 372 patients concluded that the use of the subclavian/axillary artery should only be considered when femoral vascular access is compromised (*e.g.*, due to atherosclerotic plaques) because of frequent adverse events. However, a study by Ohira *et al.*^8^ showed in 371 patients that complications such as limb ischemia, the need to change cannulation site or local infections were less frequent with axillary cannulation than in the femoral cannulation group, while showing the same results on survival, stroke, bleeding, and duration of ECMO. Two studies investigated the use of different cannulation sites for VA-ECMO with the use of concomitant IABP: a study on 11 pigs by Bělohlávek *et al.*^16^ had shown negative effects of adding IABP to ECMO on coronary flow velocity, regardless of cannulation site (femoral or axillary). Another publication in pigs by Schroeter *et al.*^17^ showed that mean arterial pressure in the anterior interventricular artery was better with anterograde flow when IABP was active compared with retrograde flow.

### Effects of Heart Rate

As in our previous publication,^9^ heart rate also has a significant influence on the benefits of IABP at VA-ECMO. For cerebral and coronary flow, the beneficial effect of IABP was greatest at a heart rate of 60 bpm, irrespective of the arterial cannulation site (Figures 4 and 5). Papaioannou *et al.*^18^ had shown that the optimal heart rate for IABP use could lie between 80 and 110 bpm, and that the maximum effect on coronary flow increase was observed at a rate <90 bpm. Xu *et al.*^19^ had shown a reduction in left carotid flow during IABP activation in patients with VA-ECMO FF and very poor cardiac function. This phenomenon fades as heart rate increases, probably because total diastolic time is reduced, thus decreasing inflation time. Chung *et al.*^20^ showed that the mean diastolic time at 60 bpm was around 600 ms (or 36 seconds/minute), compared with 300 ms at 100 bpm (or 30 seconds/minute). We observed that at 100 bpm, renal flow was less affected by IABP than at 60 bpm (Figure 6).

Our study is the first *in vitro* to investigate the concomitant use of IABP and VA-ECMO, comparing two clinically used peripheral cannulation sites. It provides new light for thought and further clarification of circulatory support devices in the treatment of drug-refractory LCOSs. Other discharge devices are available as left ventricle assisting devices such as the Impella. A recent study concluded that IABP as a left ventricular unloading device showed similar results in terms of mortality and fewer complications than Impella.^21^ A retrospective study of 52 patients by Au *et al.*^22^ also showed no advantage of Impella over IABP. In future projects, we plan to study the hemodynamic effects of this concomitant use of LVAD/VA-ECMO, and to compare *in vitro* a possible superiority between LVAD and IABP.

Our study has several limitations. First, our circuit lacks the *in vivo* dynamic autonomic regulation that would influence inotropy, heart rate, and peripheral vascular resistance, which are major determinants of cardiac output. Second, IABP timing settings were checked by an experienced clinician, but only a posteriori. Fine timing variation requires adjustments based on live curves, which could have optimized the use of the IABP during data collection.

In severe shock states, ECMO blood flow often exceeds 3 L/min. However, in our study, we assessed flow variations with a maximum native cardiac output (with the pulsatile pump) of 2 L/min and an ECMO flow capped at 3 L/min. Higher flow rates were not tested due to the physical constraints of our model, particularly the blood reservoir, which could not accommodate a total flow exceeding 5 L/min. Despite this limitation, we believe that the observed flow trends are robust and can reasonably be extrapolated linearly to scenarios with higher ECMO flows.

We acknowledged that the renal flow was underestimated (300 ml/min *vs.* 20% of the cardiac output *in vivo*) but we encountered limitations inherent to our model. Not all aortic ostia and branches were represented, and simultaneous regulation of all flows was challenging. Peripheral resistance adjustments were performed using highly sensitive metallic clamps on silicone tubing, which deformed under pressure, causing resistance variations. Once a stable flow of 300 ml/min was achieved, measurements were conducted with constant flow without modifying resistances across scenarios. Despite these constraints, we believe that the observed trends in flow variations induced by the IABP remain valid and unaffected by this underestimation.

## Conclusions

The present *in vitro* study shows that the hemodynamic effects of VA-ECMO and IABP depend on numerous parameters. In general, we found that these hemodynamic changes depend on the severity of the LCOS, the patient’s heart rate, and the site of arterial cannulation of the VA-ECMO. In cases of severe LCOS, the VA-ECMO FA configuration in combination with IABP showed the greatest benefit in terms of coronary and cerebral flow. It was also in this configuration that renal flow was most reduced. These results could provide new elements for clinical decision-making in the treatment of patients with LCOS.
